# Long‐distance dispersal in the short‐distance dispersing house sparrow (*Passer domesticus*)

**DOI:** 10.1002/ece3.11356

**Published:** 2024-04-29

**Authors:** Peter S. Ranke, Michael L. Pepke, Jørgen S. Søraker, Gabriel David, Yimen G. Araya‐Ajoy, Jonathan Wright, Ådne M. Nafstad, Bernt Rønning, Henrik Pärn, Thor Harald Ringsby, Henrik Jensen, Bernt‐Erik Sæther

**Affiliations:** ^1^ Centre for Biodiversity Dynamics (CBD), Department of Biology Norwegian University of Science and Technology (NTNU) Trondheim Norway; ^2^ BirdLife Norway Trondheim Norway; ^3^ Center for Evolutionary Hologenomics, Globe Institute, Faculty of Health and Medical Sciences University of Copenhagen Copenhagen Denmark; ^4^ Edward Grey Institute, Department of Biology University of Oxford Oxford UK; ^5^ Animal Ecology, Department of Ecology and Genetics Uppsala University Uppsala Sweden; ^6^ Department of Teacher Education Norwegian University of Science and Technology (NTNU) Trondheim Norway; ^7^ Department of Aquatic Resources (SLU Aqua) Swedish University of Agricultural Sciences Lysekil Sweden; ^8^ The Gjærevoll Centre, Department of Biology Norwegian University of Science and Technology (NTNU) Trondheim Norway

**Keywords:** capture–mark–recapture, dispersal distance, dispersal distribution, dispersal scale, genetic composition, inbreeding

## Abstract

The house sparrow (*Passer domesticus*) is a small passerine known to be highly sedentary. Throughout a 30‐year capture–mark–recapture study, we have obtained occasional reports of recoveries far outside our main metapopulation study system, documenting unusually long dispersal distances. Our records constitute the highest occurrence of long‐distance dispersal events recorded for this species in Scandinavia. Such long‐distance dispersals radically change the predicted distribution of dispersal distances and connectedness for our study metapopulation. Moreover, it reveals a much greater potential for colonization than formerly recorded for the house sparrow, which is an invasive species across four continents. These rare and occasional long‐distance dispersal events are challenging to document but may have important implications for the genetic composition of small and isolated populations and for our understanding of dispersal ecology and evolution.

## Graphical abstract

The house sparrow (*Passer domesticus*) is a small passerine known to be highly sedentary. In this paper, we present the highest occurrence of long‐distance dispersal records for this species in Scandinavia. Long‐distance dispersers may have important implications for the genetic composition of small and isolated populations.

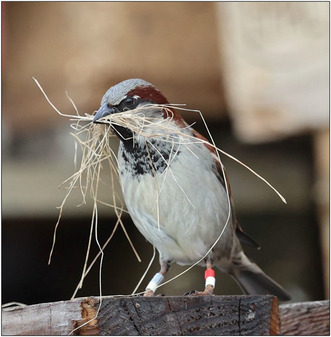

## 1 INTRODUCTION

Knowledge of the extent of long‐distance dispersal is important for predicting scale‐dependent dispersal rates within study systems, which enables predictions of population viability for small and isolated populations (Sutherland et al., 2000; Trakhtenbrot et al., 2005). It is also necessary for a better understanding of both the rate of spread of expanding and invasive populations (Neubert & Caswell, 2000) and local adaptation processes (Garant et al., 2007). The house sparrow (*Passer domesticus*) is known to be highly sedentary (Anderson, 2006; Summers‐Smith, 1988), and bird ringing records show that most house sparrows generally disperse over very short distances from their natal site (in Great Britain on average below 2 km, Paradis et al., 1998). In Finland, 90% of natal dispersal distances were less than 16 km (Kekkonen et al., 2011), in Denmark 98% were within 10 km (Bønløkke et al., 2006), and on the Faroe Islands, 99% were within 1 km (Hammer et al., 2014). However, in Denmark two records exceeded 100 km, one individual from Helgoland, Germany, to the Danish west coast (108 km), and one individual from Skåne, Sweden, to Bornholm (133 km, Bønløkke et al., 2006). In Sweden, seven records exceeded 50 km (three of which exceeded 100 km, Fransson & Hall‐Karlsson, 2008).

In Norway, the house sparrow is an abundant bird species in human‐populated areas and in agricultural landscapes across the country. It is even found in the northernmost regions (e.g., Kirkenes, Finnmark), where populations have been known to exist since the early 1900s (Collett, 1921). In our main house sparrow capture–mark–recapture study system on the Helgeland coast in northern Norway (Araya‐Ajoy et al., 2021; Ranke et al., 2021), most individuals remain sedentary (ca. 83%), and the remaining ca. 17% disperse over relatively short distances (most often 10–15 km; Pärn et al., 2009; Ranke et al., 2021; Tufto et al., 2005). However, recent advances in genetic assignment methods have revealed that in some years and on some islands as many as around 22% of recruits may have dispersed during their first year of life in our study metapopulation (Saatoglu et al., 2021). Our study metapopulation covers an area of around 1600 km^2^, spanning 18 small islands in a coastal archipelago in northern Norway. Although this study system is large compared to the usual scale of movement distances in house sparrows (Kekkonen et al., 2011; Tufto et al., 2005), it is likely that we fail to document occasional dispersal distances exceeding 50 km.

**FIGURE 1:**
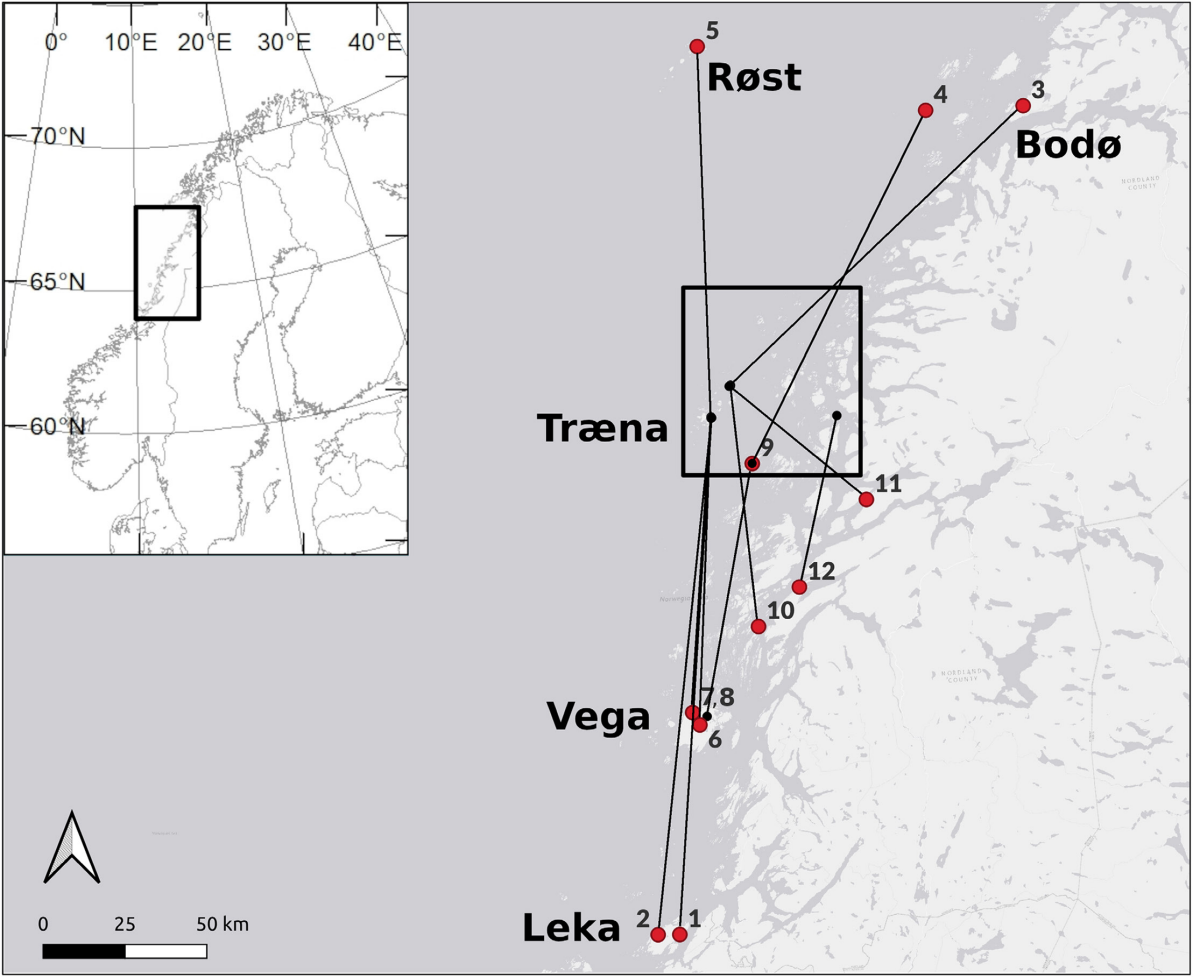
Long‐distance dispersal events of house sparrows (*Passer domesticus*) from and to the Helgeland archipelago in northern Norway during the years 1992–2022. Black dots represent ringing locations, and red dots represent the recovery sites. The black square in the main map indicates the main island archipelago metapopulation study site. Numbered identities of recoveries refer to Table 1.

During the years 1992–2022, we have in our main study metapopulation ringed a total of 25,425 individuals (16,349 nestlings and an additional 6932 fledged juveniles and 2144 recruits with unknown age). Recaptures and observations of these individuals throughout their lifetime enable us to record inter‐island movements (Ranke et al., 2021). However, additional sampling along the Norwegian coast (Jensen et al., 2013) and research we have carried out in house sparrow populations between ca. 15 and 300 km south of the main study metapopulation during the years 2001–2022 (in total ca. 11,750 house sparrows ringed; Kvalnes et al., 2017; Nafstad et al., 2023; Ranke et al., 2017, 2020; Stubberud et al., 2017), combined with regular ringing recoveries by local inhabitants (Bakken et al., 2006), allow us to explore patterns in a high number of individual house sparrow movements across long periods of time and over large geographical distances, which exceed those of our main study metapopulation (see Figure 1). Here, we aim to describe and document such long‐distance dispersal events in reference to the dispersal dynamics within a metapopulation of house sparrows. The extent of long‐distance dispersal events may have implications for dispersal distance distributions or kernels (Tufto et al., 2005), the estimation of fitness of local and dispersing individuals within spatially constrained study systems (Doligez & Pärt, 2008), the rate of immigration of novel genotypes affecting levels of inbreeding (Green & Hatchwell, 2018) and thus long‐term population viability (Sutherland et al., 2000), the transmission of infectious diseases (Ferraguti et al., 2019), and for unraveling drivers of biogeographical colonization dynamics (Pedersen et al., 2018).

## 2 METHODS

The house sparrow has been a well‐suited study species for several research fields in biology due to its sedentary and synanthropic behavior and because it naturally breeds close to human settlements (Anderson, 2006; Summers‐Smith, 1988). House sparrows have been studied in the Helgeland archipelago (66.30°–66.80° N, 12.00°–13.10° E; see black square in Figure 1) off the coast of northern Norway since 1992 (Baalsrud et al., 2014; Sæther et al., 1999). This involves research on small, fragmented populations within a metapopulation setting. The study system thus consists of small human‐inhabited islands that have historically been sustained through fishing with some subsistence farming. Today, some islands are more densely populated with village centers (situated farther off the coast; hereafter referred to as “non‐farm islands”), while others have remained scarcely populated with dairy farms (closer to the mainland; hereafter referred to as “farm islands”). Within our study metapopulation, the outer non‐farm islands show proportionally more and longer distances of natal dispersal than the inner farm islands (Ranke et al., 2021).

**FIGURE 2:**
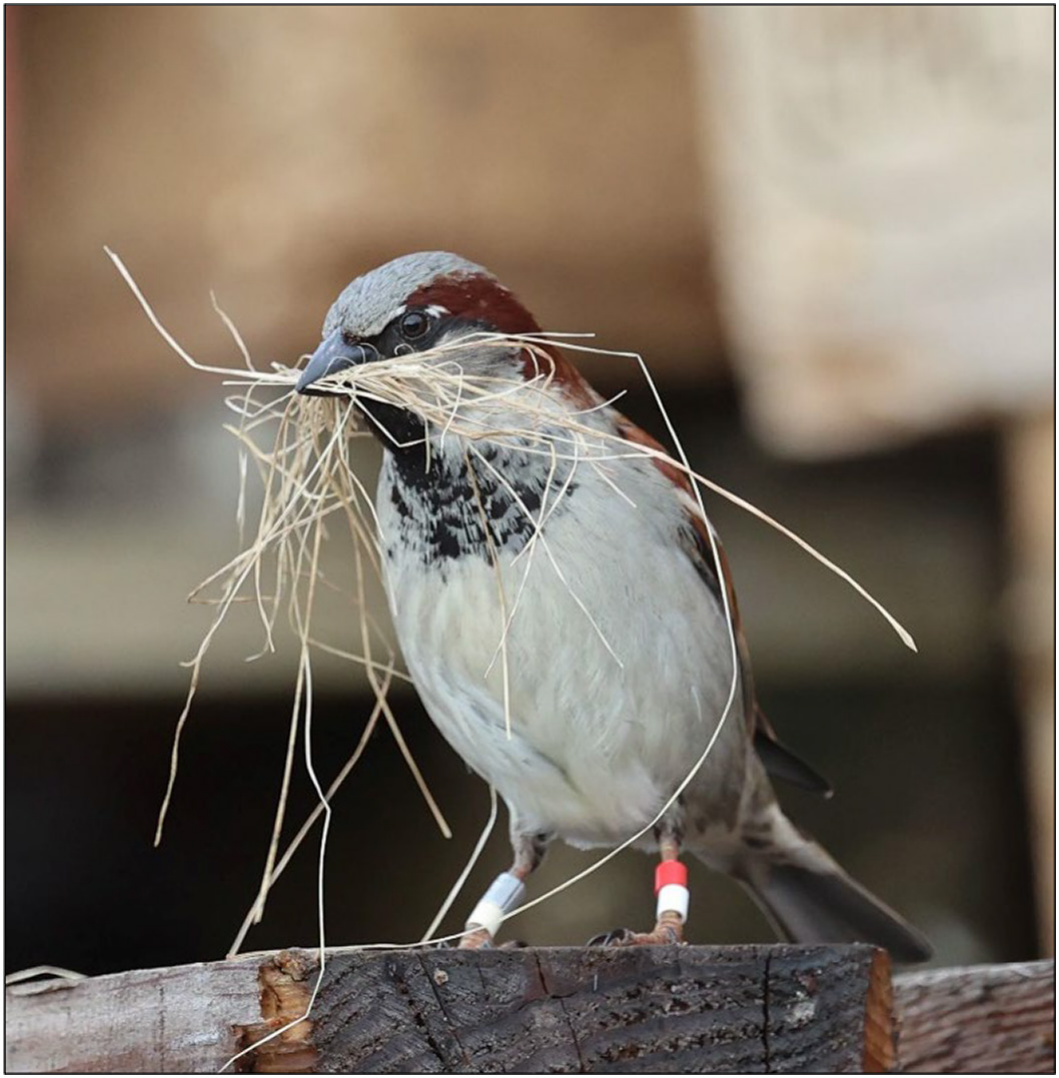
A house sparrow (*Passer domesticus*) male with a unique combination of color rings and a numbered metal ring at one of our study islands, Selvær, in the Helgeland archipelago in northern Norway. The above individual is shown carrying nest‐building materials, en route to the nest site (photo by P. S. Ranke).

Fieldwork is carried out at irregular intervals throughout the year, but almost continuously from May to mid‐August and from mid‐September to the end of October. All newly recorded individuals are given a unique combination of rings, consisting of a metal ring and three additional colored plastic rings (Figure 2). Captures using mist nets, and re‐sightings of ringed individuals, enable us to closely monitor each population throughout the season. During the breeding season, nests are searched for thoroughly on each study island, and natural nests as well as nest boxes are checked at least every 10 days. Nestlings are ringed at 11 days (5–13 days) of age. A small blood sample is collected from adults and nestlings using venipuncture from the wing, allowing us to perform genetic analyses and confirm individual sex and identity (described in Jensen et al., 2013; Saatoglu et al., 2021).

Dispersal in house sparrows occurs mainly in the autumn by juveniles (i.e., natal dispersal, Altwegg et al., 2000). In the period 1992–2014, we have recorded average dispersal rates across years and islands of around 17% from ecological data (Ranke et al., 2021) and approximately 22% based on genetic analyses (Saatoglu et al., 2021). Here, we document individual dispersal events exceeding 50 km (i.e., outside our main metapopulation study system; Table 1, Figure 1), which we would have been unable to record without a wider Ringing Scheme system of ringing recoveries.

**TABLE 1:** Overview of long‐distance dispersal events (>50 km; *n* = 12) in house sparrows (*Passer domesticus*) from or to the Helgeland archipelago, northern Norway, during the years 1992–2022.

No.	ID	Sex	Age (cy)	Date	Distance	Journey	Circumstances
1	8M31147	M	1–2	2009‐07‐01 2010‐02‐11	159	Træna Leka	Nestling Captured
2	8N13455	M	1–2	2014‐06‐29 2015‐02‐04	159	Træna Leka	Nestling Captured
3	8P14515	F	1–2	2019‐06‐15 2020‐06‐23	124	Selvær Bodø	Nestling Found dead
4	8P14582	X	1–2	2019‐06‐29 2020‐05‐18	120	Lovund Givær	Juvenile Captured
5	8N13408	M	4–5	2014‐06‐22 2018‐06‐09	113	Træna Røst	Juvenile Photographed
6	8M31109	M	1–2	2009‐06‐25 2010‐02‐19	94	Træna Vega	Nestling Captured
7	8L57742	M	1–2	2007‐05‐23 2008‐02‐24	92	Træna Vega	Nestling Captured
8	8L89915	M	1–4	2009‐05‐21 2012‐03‐07	90	Træna Vega	Nestling Captured
9	8866658	F	1–2	2002‐06‐19 2003‐05‐23	77	Vega Lovund	Nestling Captured
10	8N13261	F	1–2	2014‐05‐29 2015‐02‐23	75	Selvær‐Træna Austbø	Nestling Captured
11	8N42048	F	1–2	2017‐06‐30 2018‐04‐16	54	Selvær‐Træna Nesna	Nestling Captured
12	8M72794	F	1–2	2010‐07‐16 2011‐10‐10	52	Indre Kvarøy Sandnessjøen	Nestling Found dead

*Note*: Disperser identity (No.) corresponding to the map in Figure 1, ID (ring number, from the Ringing Scheme at Stavanger Museum), Sex (male ‘M’, female ‘F’ or unknown ‘X’), Age range in calendar years (cy; note that 1 is equal to the year of birth) at the time when dispersal occurred, Date of ringing, Distance in km, Journey from and to (separated with line shift), and Circumstances at ringing and recovery (all recovered being either captured [*n* = 9], photographed [*n* = 1] or found dead [*n* = 2]).

Dispersal distances were recorded for each individual as the shortest direct route from its natal site, either determined as the exact location of the nest where it was hatched, or measured as the geographic center of the population of house sparrows within the natal island (±500 m) to the first breeding site. For our own records, the exact location of the recovery was obtained from our database, but in other cases we received records from the Ringing Scheme (Bakken et al., 2006) with negligible lower resolution (i.e., within ±1 km). Thus, minimum distance traveled could be calculated with acceptable certainty for all dispersal events.

## 3 RESULTS AND DISCUSSION

The present study demonstrates that house sparrows may disperse over much longer distances than previously anticipated for this highly sedentary bird species, and at least up to 159 km in northern Norway. Our 12 long‐distance (>50 km) dispersal records represent a large proportion of long‐distance dispersal events recorded for house sparrows in Scandinavia (seven in Sweden, four in Denmark (excluding one Danish record of exactly 50 km), and five additional records elsewhere in Norway, see Table A1). Thus, out of a total 28 long‐distance dispersal events in Scandinavia in the past 100 years, nearly half (43%; *n* = 12) of these are recorded in the Helgeland archipelago. The number of ringed house sparrows is similar across the three countries, and our metapopulation under study constitutes about one‐third of the ringed house sparrows in Norway.

The proportion of undetected successful dispersal events cannot currently be distinguished from mortality in our study area. Moreover, the detectability outside our study area is lower than inside, and thus, many long‐distance dispersal events might remain undetected. Previous examination of dispersal within our study system revealed that 17%–22% of the recruits had dispersed (Ranke et al., 2021; Saatoglu et al., 2021), but recruitment probabilities vary between 15% and 20% (Ringsby et al., 2002; Sæther et al., 1999); thus, most juveniles are probably lost due to mortality by predation, parasite infection (Holand et al., 2014), starvation or at sea during dispersal. Out of 12 dispersal records exceeding 50 km, 10 were emigrating from and one immigrating to the outer non‐farm islands in our study system (Table 1; Figure 1), which is consistent with the generally higher levels and longer dispersal distances on average for this set of islands (Ranke et al., 2021; Saatoglu et al., 2021).

Average dispersal distances recorded in this metapopulation exceed normal dispersal distances for house sparrows in other countries (Kekkonen et al., 2011; Paradis et al., 1998) and other sites in Norway (Bakken et al., 2006). This may partly be due to the geographical structure of our study system, where individuals are forced to move across larger distances over open sea compared with more contiguous non‐coastal landscapes (Kekkonen et al., 2011; Ranke et al., 2021). This is analogous to Eurasian nuthatch (*Sitta europaea*) populations in patchy habitats in Belgium showing longer dispersal distances than populations in contiguous forest habitats (Matthysen et al., 1995). However, the number of long‐distance house sparrow dispersal events recorded here is disproportionately high, as there have been only five other house sparrow dispersal records exceeding 50 km in Norway during 1914–2022 (Bakken et al., 2006; Norwegian Ringing Scheme *pers. comm*.; see Table A1 in Appendix). The extent and duration of our fieldwork campaign, resulting in high recapture probabilities across a large geographic area (Jensen et al., 2013; Kvalnes et al., 2017; Nafstad et al., 2023; Ranke et al., 2017, 2020), have additionally contributed to documenting rare dispersal events, and our intensive field campaign contributes to about half of our documented long‐distance dispersal events. Moreover, color‐ringed individuals would be more conspicuous and easier to detect also for local inhabitants, which might additionally increase detection probabilities of individuals from our study populations.

Furthermore, we suggest that the extreme northern location and marginal environmental conditions of our study site – at the edge of the global range distribution for house sparrows – and the low density of human settlements and farms, mean that there are large distances between adjacent viable habitats. For example, house sparrows in Kenya display greater genetic variation toward the edges of their range expansion, supporting the suggestion of a higher rate of long‐distance dispersal in such areas (Schrey et al., 2014). However, similar to Schrey et al. (2014), we cannot rule out potential effects of human‐mediated dispersal (in our area by ferries, fishing boats, etc.) potentially facilitating some of these long‐distance dispersal events. We have observed sparrows on the ferries, but this is extremely rare, and so it is unclear whether ship‐assisted dispersal has any general impact on dispersal rates within our study area. Moreover, similar long‐distance dispersal events in North American house sparrows may have taken place during recent range expansion (Gibson, 2012; Schrey et al., 2011), and in some places this has included geographical landscapes similar to those of our study populations situated at high northern latitudes. In northern Norway, harsh weather conditions, including autumn storms during the period of natal dispersal, may affect wind‐assisted geographic displacement over water and across large distances (e.g., Darlington, 1938).

These types of long‐distance dispersal events highlight the potential for colonization of new habitats by house sparrows, which may be an important and hitherto overlooked part of this species success as a global colonizer and invasive species (Anderson, 2006; Hanson et al., 2020). Future studies may utilize these observations to investigate if individual phenotypic differences in, for example, morphology (Skjelseth et al., 2007), physiology (Nafstad et al., 2023; Pepke et al., 2022), or life‐history characteristics (Pärn et al., 2009; Saatoglu et al., 2024) underlie longer‐than‐expected dispersal distances (Tufto et al., 2005) in a species with very low flight efficiency (Claramunt, 2021). If dispersing individuals can successfully recruit into a breeding population, then they can offer a valuable genetic contribution to small, isolated, and often inbred populations (Dickel et al., 2021), such as these house sparrow populations habituating to island life on the northern Norwegian coast (Niskanen et al., 2020; Ranke et al., 2020). Thus, although long‐distance dispersal happens infrequently in house sparrows, long‐distance dispersal can have major implications for gene flow across large geographic areas (Garant et al., 2007), which in turn may influence the rate of inbreeding (Keller et al., 2001), population (re)colonization and spatio‐temporal population dynamics (Baalsrud et al., 2014; Billing et al., 2012; Ranke et al., 2021) and adaptive and non‐adaptive population differentiation (Aase et al., 2022; Araya‐Ajoy et al., 2019; Holand et al., 2011; Jensen et al., 2013). Furthermore, long‐distance dispersal can affect estimates of fitness of dispersers compared to non‐dispersers, because of the bias in the number of recorded offspring, especially if dispersal behavior has a heritable component (Doligez & Pärt, 2008; Saatoglu et al., 2024). This study therefore reveals the biological and methodological implications of a long tail of upper values in the distribution of dispersal distances, even in an otherwise sedentary species, and therefore the need for large‐scale longitudinal field studies quantifying these rare but important dispersal events.

## AUTHOR CONTRIBUTIONS

**Peter S. Ranke:** Conceptualization (lead); data curation (lead); visualization (lead); writing – original draft (lead); writing – review and editing (equal). **Michael L. Pepke:** Writing – review and editing (equal). **Jørgen S. Søraker:** Writing – review and editing (equal). **Gabriel David:** Writing – review and editing (equal). **Yimen G. Araya‐Ajoy:** Writing – review and editing (equal). **Jonathan Wright:** Writing – review and editing (equal). **Ådne M. Nafstad:** Writing – review and editing (equal). **Bernt Rønning:** Writing – review and editing (equal). **Henrik Pärn:** Writing – review and editing (equal). **Thor Harald Ringsby:** Writing – review and editing (equal). **Henrik Jensen:** Writing – review and editing (equal). **Bernt‐Erik Sæther:** Funding acquisition (lead); writing – review and editing (equal).

## CONFLICT OF INTEREST STATEMENT

None declared.

## Data Availability

All data are provided in Table 1, Table A1, and Figure 1.

## Appendix

**TABLE A1:** Overview of long‐distance dispersal events (>50 km; *n* = 5) for house sparrows (*Passer domesticus*) in Norway outside the Helgeland archipelago during years 1914–2022.

No.	ID	Sex	Age (cy)	Date	Distance	Journey	Circumstances
1	8N05951	M	1–2	2012‐05‐26 2013‐01‐06	102	Lauvøya Støren	Nestling Photographed
2	849776	X	1–2	1955‐07‐08 1956‐05‐21	87	Nordfjordeid Tomrefjord	Nestling Found dead
3	72412	X	1–5	1954‐08‐03 1958‐05‐09	60	Nordfjordeid Florø	Nestling Found dead
4	032459	F	1–2	1948‐06‐03 1949‐06‐18	56	Steinkjer Overhalla	Nestling Killed
5	DB84457	X	1–2	1990‐07‐26 1991‐05‐13	55	Lista Kleven, Mandal	Juvenile Killed/window

*Note*: Disperser identity (No.), ID (ring number, from a Norwegian Ringing Scheme [Stavanger Museum, Zoological Museum or Stat. Vilt. Ås]), Sex (male ‘M’, female ‘F’ or unknown ‘X’), Age range in calendar years (cy; note that 1 is equal to the year of birth) at the time when dispersal occurred, Date of ringing, Distance in km, Journey from and to (separated with line shift), and Circumstances at ringing and recovery (all recovered being either found dead [*n* = 2], killed [*n* = 2], or photographed [*n* = 1]).
